# Residues, Distributions, Sources, and Ecological Risks of OCPs in the Water from Lake Chaohu, China

**DOI:** 10.1100/2012/897697

**Published:** 2012-11-28

**Authors:** Wen-Xiu Liu, Wei He, Ning Qin, Xiang-Zhen Kong, Qi-Shuang He, Hui-Ling Ouyang, Bin Yang, Qing-Mei Wang, Chen Yang, Yu-Jiao Jiang, Wen-Jing Wu, Fu-Liu Xu

**Affiliations:** MOE Laboratory for Earth Surface Processes, College of Urban and Environmental Sciences, Peking University, Beijing 100871, China

## Abstract

The levels of 18 organochlorine pesticides (OCPs) in the water from Lake Chaohu were measured by a solid phase extraction-gas chromatography-mass spectrometer detector. The spatial and temporal distribution, possible sources, and potential ecological risks of the OCPs were analyzed. The annual mean concentration for the OCPs in Lake Chaohu was 6.99 ng/L. Aldrin, HCHs, and DDTs accounted for large proportions of the OCPs. The spatial pollution followed the order of Central Lakes > Western Lakes > Eastern Lakes and water area. The sources of the HCHs were mainly from the historical usage of lindane. DDTs were degraded under aerobic conditions, and the main sources were from the use of technical DDTs. The ecological risks of 5 OCPs were assessed by the species sensitivity distribution (SSD) method in the order of heptachlor > **γ**-HCH > p,p′-DDT > aldrin > endrin. The combining risks of all sampling sites were MS > JC > ZM > TX, and those of different species were crustaceans > fish > insects and spiders. Overall, the ecological risks of OCP contaminants on aquatic animals were very low.

## 1. Introduction

As typical persistent organic pollutants (POPs), organochlorine pesticides (OCPs) were widely used and have threatened the ecosystem and human health due to the need for pest control. There are 15 OCPs in the list produced by the Stockholm Convention on Persistent Organic Pollutants, which forbids the production and use of 22 of chemical substances, including DDT, chlordane, mirex, aldrin, dieldrin, endrin, hexachlorobenzene, heptachlor, toxaphene, *α*-HCH, *β*-HCH, lindane (*γ*-HCH), chlordecone (kepone), pentachlorobenzene, and endosulfan [1, 2]. Although these OCPs have been banned (especially DDT) and the residual levels have gradually decreased since the 1980s, OCPs can still be detected in various environmental and biological media [3–5].

OCPs can enter the water, one of the environmental media that is most vulnerable to OCP contaminants through a variety of routes, such as surface runoff and atmospheric wet and dry deposition. At present, there are residues of OCPs in the surface water including rivers, lakes, and oceans, such as the Küçük Menderes River in Turkey [6], the Ebro River in Spain [7], the Gomti River in India [8], and the section from the Sea of Japan to the Bering Sea [9]. There has also been much research on the distribution of organochlorine pesticides in the environment, such as that in the Huaihe River [10], the Pearl River [11], the Guanting Reservoir in Beijing [12], and Lake small Baiyangdian [13]. The residue concentrations in these regions were different.

**Figure 1:**
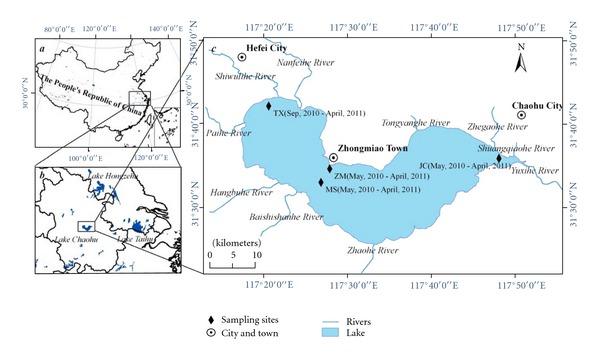
The location of Lake Chaohu and the distribution of the sampling sites.

Lake Chaohu, located in the center of Anhui Province (Figure 1), is the fifth-largest freshwater lake in China, with a water area of approximately 760 km^2^. In addition to the development of fisheries and agricultural irrigation, Lake Chaohu is also the drinking water source for the 9.6 million residents in the surrounding areas, and the water quality will affect the health and safety of the residents directly. Therefore, this study on the residual levels of the organochlorine pesticides (especially HCHs and DDTs), their spatial and temporal distributions, the source analysis, and the ecological risks will not only contribute to understanding the environmental behavior and potential hazards of persistent toxic pollutants but also provide the necessary theoretical basis for persistent toxic pollution prevention and lake environmental management.

## 2. Materials and Methods

### 2.1. Measurement of OCPs in the Water

The water samples were collected from May 2010 to February 2011 monthly, and the distribution of the sample sites is shown in Figure 1. The MS and ZM are located at 200 meters south of the Zhongmiao Temple and 200 meters east of Mushan Island, respectively; the JC and TX represent the city water intake near the Chaohu automatic monitoring station and western TangXi, 150 meters south of the intake of original waterworks, respectively.

The surface, middle, and bottom water samples were collected separately and then mixed together. In the sampling sites having depths of more than 1 meter, the water samples were collected from the surface water (0–0.15 m below the surface), the midwater (0.5–0.65 m below surface), and, the bottom water (0–0.15 m above the sediment) and mixed. In the sites having depths of less than 1 meter, the surface water and the bottom water were collected and mixed. The water samples were stored in brown glass jars that were washed with deionized water and the water samples before use. From each site, 1 liter of water was collected.

As a recovery indicator, 100 ng 1-Bromo-2-nitrobenzene was added to the water samples, which were then filtered through a glass fiber filter (ashed at 450°C for 4 h) using a peristaltic pump (80EL005, Millipore Co., USA) and a filter plate with a 142 mm diameter to remove the suspended particles. A solid phase extraction system was used to extract the filtered water samples. Before extraction, the octadecylsilane SPE cartridges (SPE, C18, 6 mL, 500 mg, Supelco, Co., USA) were first washed with 6 mL dichloromethane and conditioned with 6 mL methanol and 6 mL ultrapure water, and the cartridges were not dried before loading the samples. After the activation, the water samples were loaded using a large volume sampler (Supelco Co., USA) that was connected to the SPE vacuum manifold (Supelco Co., USA), and the cartridges were dried by vacuum pump after the extraction step. The SPE cartridges were sealed and delivered back to laboratory prior to the elution and purification.

Each cartridge was connected to an anhydrous sodium sulfate (5 g) cartridge and eluted using DCM (three times, 6 mL per elution). The extracts were concentrated to approximately 1 mL with a vacuum rotary evaporator (Eyela N-1100, Tokyo Rikakikai Co., Japan). The solvent was changed to hexane, and then the samples were again concentrated to approximately 1 mL. PCNB (pentachloronitrobenzene) was added to the sample as an internal standard. The samples were concentrated to 10 *μ*L with flowing nitrogen, transferred to micro volume inserts, and sealed until analysis. 

The samples were analyzed using an Agilent 7890A-5975C gas chromatography and mass spectrometer detector and a HP-5MS fused silica capillary column (30 m × 0.25 mm × 0.25 *μ*m, Agilent Co., USA). Helium was used as the carrier gas at a flow rate of 1 mL/min. Samples (1 *μ*L) were injected by the autosampler under a splitless mode at a temperature of 220°C. The oven temperature program was the following: 50°C for 2 min, 10°C/min to 150°C, 3°C/min for 240°C, 240°C for 5 min, 10°C/min for 300°C, and 300°C for 5 min. The ion source temperature of the mass spectrometer was 200°C, the temperature of the transfer line was 250°C, and the temperature of the quadrupole was 150°C. The compounds were quantified in the selected ion mode, and the calibration curve was quantified with the internal standard.

There were two parallel samples in each sampling site. The samples, the method blanks, and the procedure blanks were prepared in the same manner. The test for recovery and the detection limit of the method should be performed before the sample analysis (Table 1). 

### 2.2. Ecological Risk Assessment

In this study, the species sensitivity distribution (SSD) model was applied to evaluate the separate and combining ecological risks of typical OCPs, following these basic steps: (1) toxicological data acquisition and processing, (2) SSD curve construction, (3) calculation of the potentially affected fraction (PAF) to assess the ecological risk of a single pollutant, and (4) calculation of the multiple substances potentially affected fraction (msPAF) to assess the combining risks of multiple pollutants [14, 15].

#### 2.2.1. Toxicity Data Acquisition and Processing

Acute toxicity data (such as LC50 and EC50) or chronic toxicity data (NOEC) can be used to conduct an SSD curve, and in this study, acute toxicity data were used due to the lack of chronic toxicity data for OCPs. The toxicity data were collected from the ECOTOX database (http://www.epa.gov/ecotox/), and the search criteria included the LC50 endpoint, the exposure duration of less than 10 days, and the type of freshwater and tests in laboratories, and all species were considered. Because of the differences between the personnel and laboratory environment, there are many toxicity data on the same pollutant for the same species. In this study, the data point was the geometric mean of the toxicity data for the same species [16]. To understand the ecological risks to different types of freshwater organisms comprehensively, the toxicity data for the OCPs were classified into three patterns: (1) all species were not subdivided, (2) all species were subdivided into vertebrates and invertebrates, and (3) three subcategories for which the toxicity data were rich were selected, which included fish, insects and spiders, and crustaceans. According to the availability of the OCP toxicity data and the levels of exposure to the water of Lake Chaohu, this study selected five typical OCPs, which were p,p′-DDT, **γ**-HCH, heptachlor, aldrin, and endrin, to assess the ecological risks.  Table 2 shows the statistical characteristics of the toxicity data.

#### 2.2.2. SSD Curve Fitting

**Figure 2:**
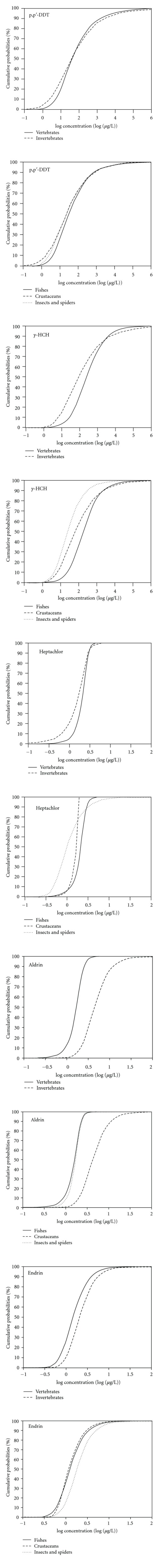
The SSD curves of typical OCPs for different species.

The basic assumption of the SSD is that the toxicity data of the pollutants can be described by a mathematical distribution and that the available toxicity data are considered as a sample from the distribution that can be used to estimate the parameters of the distribution [17]. First, the species toxicity data (e.g., LC50 or NOEC) were sorted according to the concentration values (*μ*g/L), and the cumulative probabilities of each species were calculated in accordance with the following formula [18, 19]:

(1) $$\begin{array}{c} \text{Cumulative}\;\text{Probabilities}=\frac{i}{n+1}, \end{array}$$

where *i* is the rank of species sorting and *n* is the sample size. Then, after placing the concentrations on the *X*-axis and the cumulative probabilities on the *Y*-axis in the coordinate system, these toxicity data points are marked according to the exposure concentration and cumulative probability of different organisms and fitted on the SSD curves by selecting a distribution. There are a variety of models, including parametric methods such as lognormal, log-logistic, and Burr III [20–22] and nonparametric methods such as bootstrapping [23]. At present, there is no principle for choosing the method when fitting an SSD curve because no research can prove to which specific curve form that the SSD belongs. Therefore, different researchers may choose different fitting methods [21]; for example, the researchers in the US and Europe recommended using a lognormal distribution to conduct the SSD curves, whereas others in Australia and New Zealand recommended the Burr III. Taking into account that the Burr III type requires less data and has a flexible distribution pattern that can be flexibly converted into ReWeibull and Burr III, depending on the size of the parameter values, and be conducted well using the species toxicity data [14], this study used a Burr III distribution to fit the SSD curves. In this paper, the software BurrliOZ, which was designed by Australia's Commonwealth Scientific and Industrial Research Organization (CSIRO) [24], was employed to fit the SSD curves and calculate the relevant parameters. Five OCP SSD curves for vertebrates, invertebrates, fish, crustaceans, and insects and spiders are shown in Figure 2.

#### 2.2.3. Calculation of the Single Pollutant's PAF

The PAF of the single pollutant can be calculated by the following Burr III equation:

(2) $$\begin{array}{c} F\left(x\right)=\frac{1}{{\left[1+{\left(b/x\right)}^{c}\right]}^{k}}, \end{array}$$

where *x* is the concentration of the pollutant (*μ*g/L) in the environment and *b*, *c*, and *k* are the three parameters of the model (the same as below). When *k* tends to infinity, the Burr III distribution model transforms into a ReWeibull distribution model:

(3) $$\begin{array}{c} F\left(x\right)=\exp\left(-\frac{b}{x^{c}}\right). \end{array}$$

When *c* tends to infinity, it transforms into a RePareto distribution:

(4) $$\begin{array}{c} F\left(x\right)={\left(\frac{x}{x_{0}}\right)}^{\theta },\;I\left\{x\leq x_{0}\right\}\;\;\left(x_{0},\theta >0\right). \end{array}$$

The parameters are calculated by the BurrliOZ program. When *k* is greater than 100 or *c* is greater than 80, the software will use ReWeibull or RePareto to calculate the relevant parameters automatically. The fitting parameters for p,p*'*-DDT, *γ*-HCH, heptachlor, aldrin, and endrin are given in Table 3.

#### 2.2.4. The Calculation of msPAF

The advantage of the SSD is that the msPAF can be calculated and consequently the combining ecological risks of multiple pollutants can be evaluated. According to the toxic mode of action (TMoA) by different pollutants, the msPAF was calculated using concentration addition or response addition [25]. In this study, the TMoAs of the five OCPs were different, and thus the response addition was adopted. The equation is as follows:

(5) $$\begin{array}{c} \text{msPAF}=1-\left(1-\text{PA}{\text{F}}_{1}\right)\left(1-\text{PA}{\text{F}}_{2}\right)\cdots \left(1-\text{PA}{\text{F}}_{n}\right). \end{array}$$

## 3. Results and Discussion

### 3.1. The Residues of OCPs in the Water

**Figure 3:**
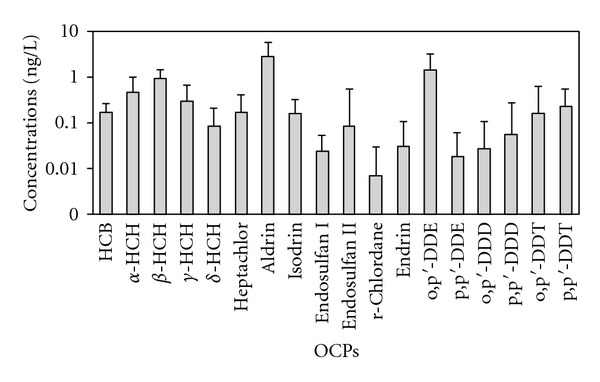
Annual mean concentrations of 18 OCPs in the water from Lake Chaohu.

Eighteen OCPs were found in the water from Lake Chaohu (Table 4), which were the following: HCH isomers (*α*-, *β*-, *γ*-, and *δ*-HCH), DDT and its metabolites (o,p′-, p,p′-DDE, DDT and DDD), heptachlor, hexachlorobenzene (HCB), aldrin, isodrin, endosulfan isomers (endosulfan I, endosulfan II), *γ*-chlordane, and endrin. The annual mean concentration of the region's total OCPs was 6.99 ng/L, and the arithmetic mean was 7.14 ± 4.19 ng/L. The detection rates of aldrin, HCB, *α*-HCH, *β*-HCH, and *γ*-HCH were 100%, while the rates of *γ*-chlordane and endrin were less than 50%; the rates of the other pollutants ranged from 64.86% to 97.3%. The residual level of aldrin (2.83 ± 2.87 ng/L) was the highest, followed by the DDTs (1.91 ± 1.92 ng/L) and the HCHs (1.76 ± 1.54 ng/L); together, these residual levels accounted for 91% of the total OCPs. The residual levels of the pollutants are illustrated in Figure 3.

Compared with other studies, the level of aldrin in Lake Chaohu was lower than that in the Pearl River artery estuary during the low flow season (4.17 ± 3.07 ng/L) [11], the Karst Subterranean River in Liuzhou (9.22 ± 1.90 ng/L) [26], and the Kucuk Menderes River in Turkey (17–1790 ng/L) [6], higher than that in the Changsha section of the Xiangjiang River (0.22–0.51 ng/L) [27] and the Wuhan section of the Yangtze River (1.88 ng/L) [28], and comparable with that in the Huaxi River in Guizhou (2.079 ng/L) [29] and the Guanting Reservoir in Beijing (2.26 ± 2.84 ng/L) [30]. The levels of HCHs were similar to those in Lake Baiyangdian (2.1 ± 0.8 ng/L) [31], considerably lower than those in the Qiantang River in Zhejiang (33.07 ± 14.64 ng/L) [32], the Chiu-lung River in Fujian (71.1 ± 85.5 ng/L) [33], and the Kucuk Menderes River in Turkey (187–337 ng/L) [6], and higher than those in Meiliang Bay in Lake Taihu (>0.4 ng/L) [34], Lake Co Ngoin in Tibet (0.3 ng/L) [35], and Lake Baikal in Russia (0.056–0.96 ng/L) [36]. The concentrations of DDTs were also at low levels, which were roughly equal to those in the Nanjing section of the Yangtze River (1.57–1.79 ng/L) [37] and lower than those in the Guanting Reservoir (3.71–16.03 ng/L) [38], the Huangpu River (3.83–20.90 [11.97] ng/L) [39], the Pearl River artery estuary during the low flow season (5.85–9.53 ng/L) [11], the Kucuk Menderes River in Turkey (ND-120 ng/L) [6], and the Lake Baikal in Russia (ND-0.015 *μ*g/L) [36].

### 3.2. The Spatial and Temporal Distribution of OCPs in the Water

**Figure 4:**
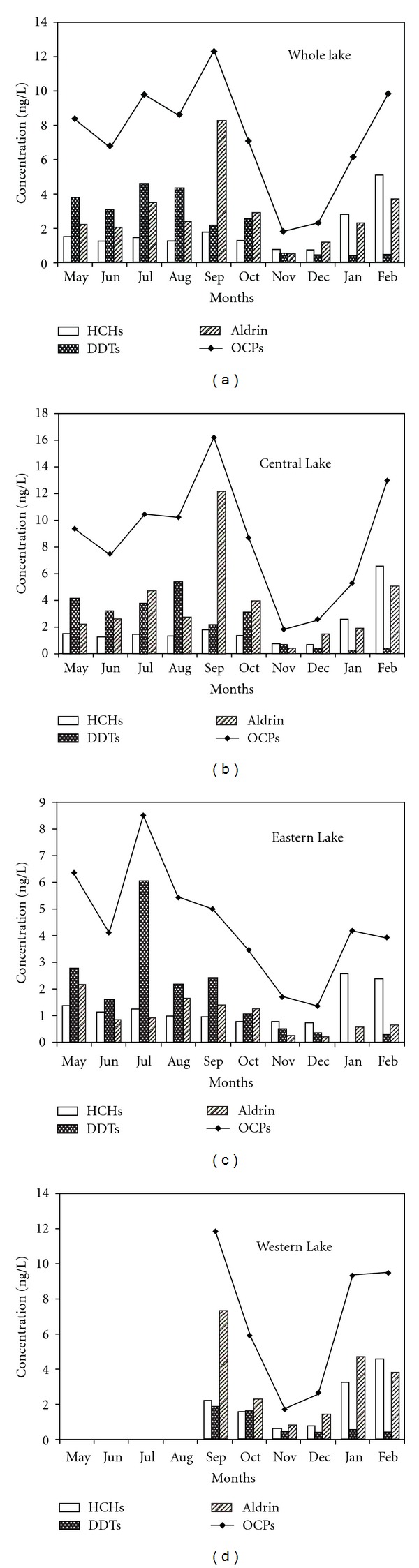
The temporal and spatial variation of OCPs in the water from Lake Chaohu.

The changes in the concentrations of the total OCPs and the three main pollutants (HCHs, DDTs, and aldrin) in Lake Chaohu and the three subregions from May 2010 to February 2011 are shown in Figure 4. There were similar trends for the OCPs over time both in the entire lake and in the Central Lake. The OCP levels increased jaggedly from May to September, and the peak was in September. Then, the residues declined rapidly, reached the bottom in November, and rose again from December to February. The trend in the Western Lake from September to February was the same, but the trend in the Eastern Lake was different. One of the main causes was that the concentrations of DDT in July were excessive, resulting in the higher OCPs from the Eastern Lake in July than that in the other months. There was presumably a temporary point source pollution in July. Moreover, the high values of aldrin both in the Western and the Central Lake in September, which were not observed in the Eastern Lake, made the overall trends of the Eastern Lake different from the other subregions.

Ten months were divided into four seasons, with spring just using the data of May as a reference. The concentrations of HCHs in the four seasons were 1.44 ng/L, 1.25 ng/L, 1.19 ng/L, and 2.81 ng/L, and the concentrations of DDTs were 3.61 ng/L, 3.75 ng/L, 1.53 ng/L, and 0.24 ng/L. The variable trends of the HCHs and the DDTs were similar except during winter, and the concentrations were higher in spring and summer than in autumn. The levels of HCHs in winter were greater than those in any other season, but the levels of DDTs were the opposite and with an order of magnitude lower in winter than in the other seasons. The possible reasons for this phenomenon included water changes and the use of related pesticides. Beginning in June, the input amount of water from Lake Chaohu was higher than the output amount, reaching the highest level in July and August. After September, the output amount of water was greater than the input, and the water of Lake Chaohu was gradually reduced. On the one hand, the increase in water diluted the pollutants in the lake, and on the other hand, new pollutants were added to the lake from the area along the river. Furthermore, the use of OCPs around the lake would result in an increase in the OCP residues in spring and summer, when there are more agricultural activities. Additionally, other technical products that include HCHs or DDTs may result in this irregular seasonal variation.

Seasonal differences in the remaining pollutants were analyzed as follows: the seasonal trends of hexachlorobenzene and heptachlor, which were similar to those of HCHs, were the highest residues in winter; the residue of aldrin was at a high concentration, but the seasonal variation was inconspicuous; the pollution of isodrin and *γ*-chlordane was severe in summer while the concentrations of endosulfan and endrin had high values in spring. These values may have certain relationships with the application characteristics of these pollutants in general without uniform trends.

Based on the spatial distribution, the sampling site JC represented the Eastern Lake and its water source areas, MS and ZM represented the Central Lake and the lakeside area of the Zhongmiao Temple, and TX represented the Western Lake region, which was near the region of the water intake. The data in TX just included September 2010 to February 2011. To ensure the comparability among the sampling sites, the monitoring data of the other three sites were also selected from this period (Table 5). The concentration of the OCPs was 3.33 ng/L from the Eastern Lake, 7.56 ng/L from the Central Lake, and 6.83 ng/L from the Western Lake. The pollution levels, from heavy to light, followed the order of Central Lake > Western Lake > Eastern Lake and the water source area, and the levels of OCPs in the Western and Central Lakes were more than twice those in the Eastern Lake and the water source area. The main pollutants in each region of the lake were different. The main pollutants were HCHs and DDTs in the Eastern Lake and the water source area and aldrin in the Western Lake and the Central Lake in addition to HCHs and DDTs. Because of fewer sampling sites, the spatial differences they reflected may be influenced by the environment around the sites. There was an unpopulated region near the site of JC, whereas the relatively dense residential areas were located near the sites of ZM and MS. The life or industrial emissions were also one of the factors that led to the high pollution levels of the lake.

### 3.3. The Composition and Source of the OCPs in the Water

#### 3.3.1. The Composition of the OCPs in the Water

**Figure 5:**
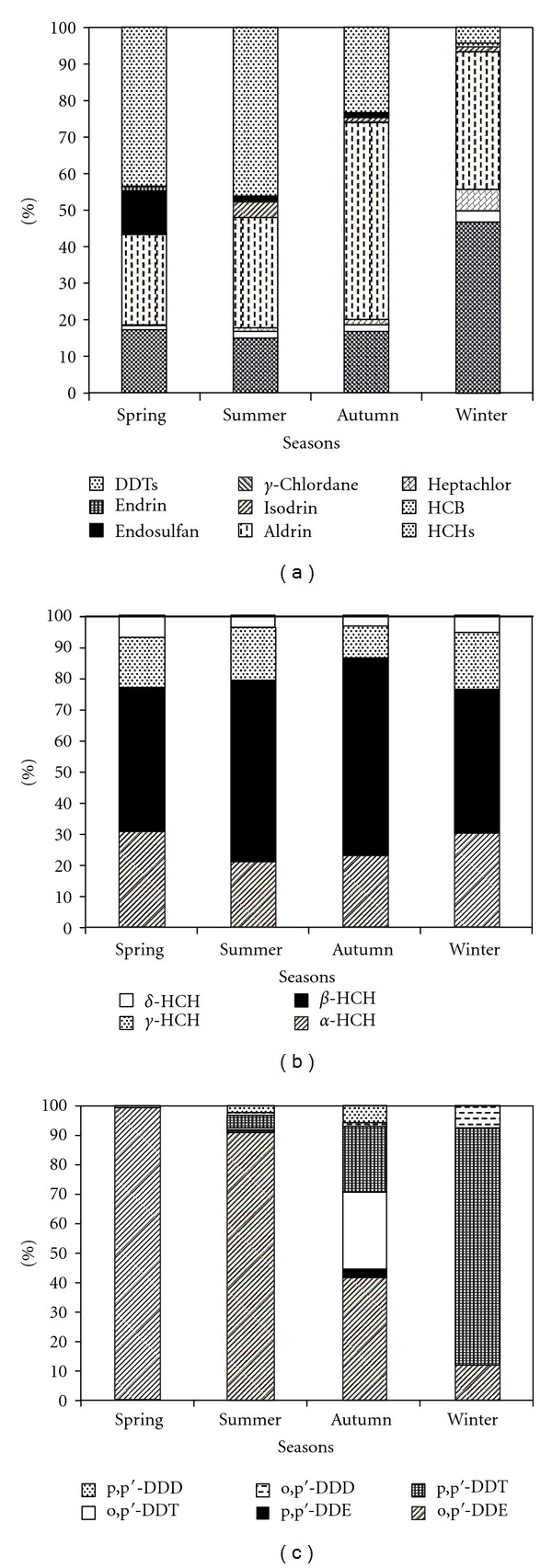
Seasonal changes of the composition of (a) OCPs, (b) HCHs, and (c) DDTs in the water.

The compositions of the OCPs, particularly the HCHs and DDTs, are shown in Figures 5(a), 5(b), and 5(c).  Figure 5(a) shows that a greater than high proportion of the OCPs (85%) was shared by aldrin, HCHs, and DDTs in the water. The level of aldrin was the highest, accounting for 54.04% in autumn and 24.94% to 37.66% in the other seasons. The highest levels of HCHs were observed (46.43%) in winter, with seasonal HCHs being the main pollutants and the levels being approximately 15% in the other three seasons. In contrast, the level of DDTs was the lowest in winter at 4.32% and higher than the other pollutants in spring and summer at 43.54% and 46.40%, respectively.

As shown in Figure 5(b), *β*-HCH was the main HCH isomer in each season, ranging from 46.20% to 63.44%, followed by *α*-HCH (20.88%–30.84%). There were no significant seasonal differences between the HCH isomers. The levels of *γ*-HCH and *δ*-HCH were relatively lower, ranging from 10.60% to 18.56% and 3.20% to 7.29%, respectively.

Figure 5(c) illustrates that o, p*'*-DDE occupied more than 90% of the DDTs in spring and summer. In autumn o, p*'*-DDE accounted for 41.39%, and o, p*'*-DDT and p, p*'*-DDT, the two other isomers of DDT, accounted for 26.31% and 22.22%, respectively. The major pollutant in the winter was p, p*'*-DDT (79.87%), whereas o, p*'*-DDE accounted for only 11.50%, and the proportion of the remaining isomers was less than 8% collectively.

#### 3.3.2. Source Identification of HCHs and DDTs

**Figure 6:**
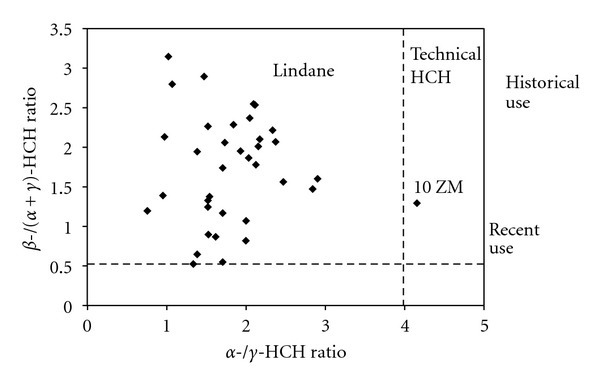
The identification of HCHs sources in the water from Lake Chaohu.

The HCH residues in the environment may come from the early use of the technical HCH or lindane and/or recent input, which can be identified according to their proportions, such as the *α*-/*γ*-HCH ratio or the *β*-/(*α* + *γ*)-HCH ratio. Technical HCH consists of 60–70%  *α*-HCH, 5–12%  *β*-HCH, and 10–15%  *γ*-HCH, with an *α*-/*γ*-HCH ratio of approximately 4–7 and a *β*-/(*α* + *γ*)-HCH  ratio of approximately 0.06–0.17. For lindane, which contains more than 99%  *γ*-HCH, the *α*-/*γ*-HCH ratio is less than 0.1 and the *β*-/(*α* + *γ*)-HCH is less than 0.06. Because of the high vapor pressures, *α*-HCH is the main isomer in the air and could be transported for long distances. Hence, the *α*-/*γ*-HCH ratio can be used to identify the source of the HCHs [36, 40]. If the *α*-/*γ*-HCH ratio is between 4 and 7, the source of the HCH may be from an industrial product, while the ratio for lindane is less than 4 [41]. *β*-HCH is the major isomer in water, soil, and sediment because of its stable physical and chemical properties. Therefore, the *β*-/(*α* + *γ*)-HCH ratio can be used to identify the history of the HCH use. The high ratio indicates the source of the historical use of technical HCH or lindane [42]. However, there is no acknowledged ratio threshold to illustrate either the historical use or the recent input. Based on the references from other studies [43], 0.5 was used as a threshold. When the *β*-/(*α* + *γ*)-HCH ratio is less than 0.5, a recent use of lindane or an atmospheric source for the input exists, and when the ratio is greater than or equal to 0.5, HCH comes from the historical use of technical HCH or lindane. According to the analysis above, we can illustrate the source of the HCH in the graph with the ratios as the axes (Figure 6).

Figure 6 shows that the *α*-/*γ*-HCH ratios of the sampling sites from May 2010 to February 2011 ranged from 0.78 to 4.16 and that only the ratio of the Zhongmiao Temple in October 2010 was greater than 4. *β*-HCH accounted for a high proportion of the total HCHs, and the *β*-/(*α* + *γ*)-HCH ratios of all sites were greater than 0.5. These observations indicated that the sources of the HCHs were mainly from the historical use of lindane after a period of degradation. 

The sources of the DDTs can be identified by analyzing their composition in the environment. Technical DDT contains approximately 14 compounds, including 75% p, p*'*-DDT and 15% o, p*'*-DDT, with the o, p*'*-/p, p*'*-DDT ratio being approximately 0.2. dicofol, a substitute for DDT that contained considerable impurities of DDTs, was widely used after the prohibition of technical DDT in 1983. o, p*'*-DDT is the major DDT impurity, and the o, p*'*-/p, p*'*-DDT ratio is 7 ± 2. A high ratio in the environment is considered to indicate pollution by dicofol [44], and a ratio of 0.2 indicates that technical DDT is the main source. Otherwise, the relative proportions of the DDT metabolites can be used to identify the source. In the environment, DDT can be degraded to DDE and DDD, and the percentage of DDT will decrease as DDE and DDD increase over time [45]. Therefore, the DDT/(DDE + DDD) ratio can indicate when the DDT was used. New inputs are indicated when the ratio is greater than or equal to 1, and historical use is indicated when the ratio is less than 1. Because DDT will be metabolized into DDE under aerobic conditions and DDD under anaerobic conditions, the DDD/DDE ratio can be used to estimate the metabolic environment of DDT. The condition is anaerobic when the ratio is greater than 1 and aerobic when the ratio is less than 1 [46, 47]. According to the analysis above, the DDT triangular graph can indicate the historical use and metabolic environment of DDT [48]. The chart with the o, p*'*-/p, p*'*-DDT and DDT/(DDE + DDD) ratios as axes can illustrate the source and use history of DDT [46].

**Figure 7:**
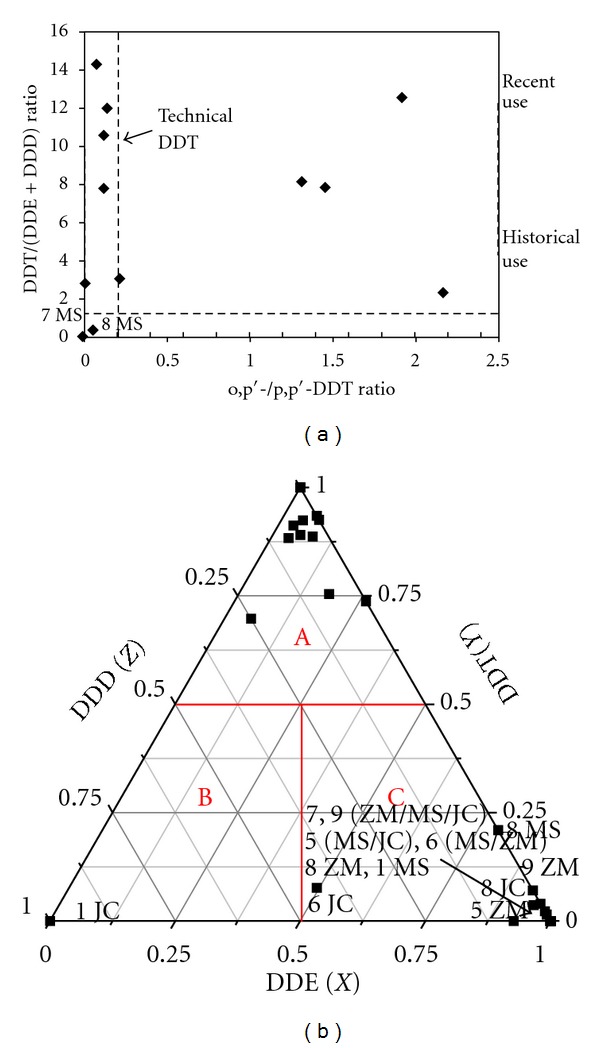
The identification of the DDT sources in the water from Lake Chaohu.

Figure 7 shows that there were 12 samples without DDT in the 36 samples in which DDTs were detected, and the o, p*'*-/p, p*'*-DDT ratios of the remaining samples ranged from 0 to 2.17, except the two samples at the JC site in June and August. These results indicated that the detectable DDTs were derived from technical DDT, while the use of dicofol made less contribution to the concentrations of DDT in the water from Lake Chaohu, which was affected near the JC site. The DDT/(DDD + DDE) ratios were less than 1 from May to September, ranging from 0 to 0.11 and increased rapidly from October, ranging from 1.10 to 13.40. On the one hand, the degradation of DDT in spring and summer was relatively significant, and on the other hand, there were new inputs in autumn and winter because the ratio was greater than 1. In addition, the low detectable rate of DDD (20.83%) indicated that the metabolic environment was aerobic, which is associated with the higher oxygen content of surface water.

### 3.4. The Ecological Risks of OCPs in Water

The SSD model was employed to assess the ecological risks for all species at four sampling sites. The average and maximum ecological risks are given in Tables 6 and 7, respectively. By comparing the mean values, the ecological risk of site MS, where the pollution of p, p*'*-DDT and aldrin was heavy, was slightly higher than those of the other sites. The potential risk of *γ*-HCH at site TX was relatively higher, while at sites JC and ZM, the risks of heptachlor and isodrin were higher. In 5 OCPs, the ecological risk of heptachlor was the highest, followed by *γ*-HCH, p, p*'*-DDT, aldrin, and endrin. However, Tables 6 and 7 indicate that the potential risks of the OCPs for all species at the four sites were very low, ranging from 7.885 × 10^−28^ to 1.639 × 10^−8^. The maximum risk probability of a single pollutant was less than 10^−7^. Comparing by species, the risks of p, p*'*-DDT and heptachlor for vertebrates were less than those for invertebrates, and the risks of the other three pollutants for vertebrates were higher. For further classification of the three subcategories, the risk of p, p*'*-DDT for crustaceans was 10^−7^, which was the highest, whereas the risk of p, p*'*-DDT was mostly harmless for fish and insects and spiders. The risk of *γ*-HCH was highest for fish (10^−8^) and was up to 10^−16^ for insects and spiders and less for crustaceans. Heptachlor had no risk for insects and spiders, but its risk for fish was two orders of magnitude higher than those for crustaceans, at 10^−12^ and 10^−14^, respectively. The risk of aldrin, and endrin was ranked as followed: fish > insects and spiders ≫ crustaceans. The risk of aldrin for fish was up to 10^−7^, whereas endrin generally had a low risk.

The results of the combining ecological risk of each site are shown in Table 8. The mean combining ecological risk probability of each site for all species was approximately 10^−10^, following the order of MS > JC > ZM > TX. The site of the highest combining risk was MS in February (1.652 × 10^−10^). A species-by-species comparison revealed that the potential combining ecological risk probability for invertebrates was 10^−6^ at the MS site in February, which was higher than that for vertebrates. Among the three subcategories, the probability of the combining ecological risks was ranked as crustaceans > fish > insects and spiders, with the maximum probability being close to 10^−6^at the MS and ZM sites. Nevertheless, the risk was actually very low because of its order of magnitude, and the pollutants had little influence on aquatic organisms. Overall, the ecological risk of OCPs for aquatic organisms in Lake Chaohu was very low.

## 4. Conclusions

1. The annual mean concentration of the total OCPs in the water from Lake Chaohu was 6.99 ng/L. The level of the total HCHs was 1.76 ng/L, which was the highest in winter, and the level of the total DDTs was 1.91 ng/L, which was higher in spring and summer than that in autumn and winter. The spatial pollutions followed from heavy to light as follows: Central Lakes > Western Lakes > Eastern Lakes and water resource district. The residues of the HCHs and DDTs were lower compared with those from other studies.
2. Aldrin, HCHs, and DDTs accounted for the majority of the OCPs, and their peak values appeared in the autumn, winter, and spring and summer, respectively. In each season, *β*-HCH was the main HCH isomer, followed by *α*-HCH, and there were no significant seasonal differences between the two. The main metabolite of DDT was o, p*'*-DDE in the spring and summer, there were two additional isomers of DDT in autumn, and p,p′-DDT was the major metabolite in winter.
3. The sources of the HCHs were mainly from the historical usage of lindane after a period of degradation. The DDTs were degraded under aerobic conditions, and the main sources were from the use of technical DDTs. The concentration of the DDTs was slightly influenced by the use of dicofol. In spring and summer, the degradation was relatively significant, but there were new DDT inputs in autumn and winter.
4. The ecological risks of 5 OCPs were assessed by the species sensitivity distribution (SSD) method in the following order: heptachlor > *γ*-HCH > p,p′-DDT > aldrin > endrin. The combining risks of all the sampling sites in decreasing order were as follows: MS > JC > ZM > TX. The combining ecological risks of different species were in the order: crustacean > fish > insects and spiders. Overall, the ecological risks of OCPs contaminants on aquatic animals were very low.

## Figures and Tables

**Table 1:** The method recoveries and the instrument detection limits.

Pollutants	Recovery %	Detection limit (ng/mL)
*α*-HCH	100.4	0.05
*β*-HCH	99.1	0.05
*γ*-HCH	88.7	0.06
*δ*-HCH	90.3	0.06
o,p′-DDE	87.1	0.2
p,p′-DDE	60.4	0.18
o,p′-DDD	80.1	0.06
p,p′-DDD	84.2	0.59
o,p′-DDT	66.4	1.2
p,p′-DDT	84.6	0.07
HCB	54.0	0.01
Heptachlor	68.0	0.1
Aldrin	71.6	0.07
Isodrin	74.7	0.03
*γ*-chlordane	66.6	0.1
Endosulfan I	89.4	0.06
Endosulfan II	95.7	0.05
Endrin	107.4	0.47

**Table 2:** The statistical characteristics of the log-transformed toxicity data for typical OCPs (*μ*g/L).

	p,p′-DDT			*γ*-HCH			Heptachlor		
	Numbers	Mean	SD	Numbers	Mean	SD	Numbers	Mean	SD
All species	151	1.782	1.148	122	2.323	1.068	48	2.08	1.11
Vertebrates	62	1.802	1.083	60	2.475	0.896	32	2.11	0.65
Invertebrates	89	1.769	1.196	62	2.175	1.201	16	1.79	0.93
Fishes	57	1.678	1.022	54	2.352	0.854	31	2.09	0.65
Crustaceans	28	1.496	1.127	20	2.048	1.151	8	1.67	0.48
Insects and spiders	50	1.516	0.939	28	1.509	0.663	6	1.48	1.14

	Aldrin			Endrin					
	Numbers	Mean	SD	Numbers	Mean	SD			

All species	55	2.08	1.11	83	83	83			
Vertebrates	31	1.72	0.66	46	46	46			
Invertebrates	24	2.54	1.39	37	37	37			
Fishes	29	1.64	0.58	40	40	40			
Crustaceans	13	2.59	1.71	10	10	10			
Insects and spiders	6	1.71	0.55	21	21	21			

SD = Standard deviation.

**Table 3:** The parameters of SSD curves calculated by BurrliOZ.

	p,p′-DDT				Lindane (*γ*-HCH)			
	Fitted curve	Parameters and values			Fitted curve	Parameters and values		
All species	Burr III	0.082(*b*)	0.489(*c*)	14.626(*k*)	Burr III	2.519(*b*)	0.515(*c*)	6.043(*k*)
Vertebrates	ReWeibull	5.146(*b*)	0.541(*c*)		Burr III	58.638(*b*)	0.708(*c*)	2.259(*k*)
Invertebrates	Burr III	0.146(*b*)	0.468(*c*)	9.786(*k*)	ReWeibull	5.450(*b*)	0.456(*c*)	
Fishes	ReWeibull	5.365(*b*)	0.593(*c*)		Burr III	57.899(*b*)	0.784(*c*)	2.085(*k*)
Crustaceans	Burr III	1.960(*b*)	0.577(*c*)	3.214(*k*)	ReWeibull	6.430(*b*)	0.526(*c*)	
Insects and spiders	ReWeibull	3.906(*a*)	0.551(*b*)		Burr III	1.560(*b*)	0.780(*c*)	6.655(*k*)

	Heptachlor				Aldrin			
	Fitted curve	Parameters and values			Fitted curve	Parameters and values		

All species	Burr III	2.704(*b*)	8.188(*c*)	0.280(*k*)	Burr III	1.860(*b*)	2.000(*c*)	3.000(*k*)
Vertebrates	Burr III	2.614(*a*)	8.839(*b*)	0.357(*k*)	Burr III	2.086(*b*)	6.654(*c*)	0.413(*k*)
Invertebrates	Burr III	2.586(*b*)	5.919(*c*)	0.284(*k*)	Burr III	2.230(*b*)	2.000(*c*)	3.000(*k*)
Fishes	Burr III	2.490(*b*)	7.902(*c*)	0.429(*k*)	Burr III	2.042(*b*)	8.036(*c*)	0.343(*k*)
Crustaceans	RePareto	2.000(*x*_0_)	4.093(*θ*)		Burr III	2.180(*b*)	2.000(*c*)	3.000(*k*)
Insects and spiders	ReWeibull	0.699(*b*)	1.636(*c*)		Burr III	1.956(*b*)	7.174(*c*)	0.521(*k*)

	Endrin			
	Fitted curve	Parameters and values			

All species	Burr III	0.987(*b*)	2.000(*c*)	3.000(*k*)				
Vertebrates	Burr III	0.724(*b*)	2.000(*c*)	3.000(*k*)				
Invertebrates	Burr III	1.041(*b*)	2.000(*c*)	3.000(*k*)				
Fishes	Burr III	0.634(*b*)	2.000(*c*)	3.000(*k*)				
Crustaceans	ReWeibull	0.957(*b*)	2.011(*c*)					
Insects and spiders	Burr III	0.924(*b*)	2.000(*c*)	3.000(*k*)				

The letter in parentheses mean the parameters *b*, *c*, *k*, *x*_0_, and *θ*.

**Table 4:** The residual levels of OCPs in the water from Lake Chaohu (ng/L).

	SD	Maximum	Minimum	Arithmetic mean	Geometric mean	Detection rate
*α*-HCH	0.53	2.40	0.11	0.47	0.33	100.00%
*β*-HCH	0.51	2.19	0.36	0.92	0.80	100.00%
*γ*-HCH	0.38	1.77	0.06	0.29	0.19	100.00%
*δ*-HCH	0.13	0.60	N.D.	0.08	0.06	83.78%
HCHs	1.45	6.92	0.55	1.76	1.41	100.00%
o,p′-DDE	1.93	7.03	N.D.	1.42	1.01	62.16%
p,p′-DDE	0.04	0.16	N.D.	0.02	0.03	32.43%
o,p′-DDD	0.08	0.38	N.D.	0.03	0.18	16.22%
p,p′-DDD	0.22	1.06	N.D.	0.06	0.07	24.32%
o,p′-DDT	0.46	2.32	N.D.	0.16	0.15	35.14%
p,p′-DDT	0.30	1.15	N.D.	0.23	0.30	62.16%
DDTs	1.92	7.03	N.D.	1.91	1.10	97.30%
HCB	0.08	0.35	0.06	0.17	0.15	100.00%
Heptachlor	0.25	1.09	N.D.	0.17	0.15	64.86%
Aldrin	2.87	12.22	0.15	2.83	1.76	100.00%
Isodrin	0.17	0.63	N.D.	0.16	0.12	91.89%
*γ*-chlodane	0.02	0.14	N.D.	0.01	0.01	32.43%
Endosulfan I	0.03	0.15	N.D.	0.02	0.03	62.16%
Endosulfan II	0.45	2.70	N.D.	0.09	0.01	48.65%
Endosulfan	0.46	2.80	N.D.	0.10	0.30	86.26%
Endrin	0.08	0.37	N.D.	0.03	0.08	27.03%

**Table 5:** The spatial distributions of OCPs in the water from September 2010 to February 2011 (ng/L).

Pollutants	MS	ZM	JC	TX
HCB	0.16	0.17	0.15	0.20
HCHs	2.30	2.13	1.36	2.14
DDTs	0.87	1.01	0.78	0.78
Heptachlor	0.29	0.23	0.22	0.14
Aldrin	3.90	3.84	0.68	3.36
Isodrin	0.08	0.07	0.11	0.09
*γ*-chlordane	0.00	0.00	0.00	0.00
Endosulfan	0.03	0.02	0.03	0.10
Endrin	0.01	0.01	0.01	0.03
OCPs	7.65	7.48	3.33	6.83

**Table 6:** The spatial variation of the mean ecological risk of typical OCPs (PAF).

Pollutant	Site	Mean value (*μ*g/L)	PAF					
			All species	Vertebrates	Invertebrates	Fishes	Crustaceans	Insects and spiders
p,p′-DDT	MS	3.556*E* − 4	4.692*E* − 18	7.440*E* − 165	6.083*E* − 13	2.494*E* − 259	1.128*E* − 07	1.329*E* − 135
	ZM	2.897*E* − 4	1.187*E* − 18	4.218*E* − 184	2.503*E* − 13	9.462*E* − 293	7.730*E* − 08	9.952*E* − 152
	JC	2.237*E* − 4	2.072*E* − 19	1.247*E* − 211	8.114*E* − 14	0.000*E* + 00	4.799*E* − 08	7.572*E* − 175
	TX	3.470*E* − 4	3.983*E* − 18	4.823*E* − 167	5.472*E* − 13	4.123*E* − 263	1.078*E* − 07	1.957*E* − 137

*γ*-HCH	MS	1.940*E* − 4	1.508*E* − 13	1.717*E* − 09	2.159*E* − 117	1.123*E* − 09	4.103*E* − 251	5.312*E* − 21
	ZM	1.911*E* − 4	1.440*E* − 13	1.676*E* − 09	3.391*E* − 118	1.096*E* − 09	4.184*E* − 253	4.913*E* − 21
	JC	1.555*E* − 4	7.615*E* − 14	1.205*E* − 09	8.951*E* − 130	7.825*E* − 10	5.199*E* − 282	1.686*E* − 21
	TX	2.282*E* − 4	2.490*E* − 13	2.226*E* − 09	4.571*E* − 109	1.465*E* − 09	1.286*E* − 230	1.233*E* − 20

Heptachlor	MS	1.436*E* − 4	1.582*E* − 10	3.606*E* − 14	7.024*E* − 08	4.264*E* − 15	1.094*E* − 17	0.000*E* + 00
	ZM	1.603*E* − 4	2.036*E* − 10	5.102*E* − 14	8.450*E* − 08	6.191*E* − 15	1.717*E* − 17	0.000*E* + 00
	JC	2.039*E* − 4	3.535*E* − 10	1.090*E* − 13	1.266*E* − 07	1.400*E* − 14	4.595*E* − 17	0.000*E* + 00
	TX	1.176*E* − 4	1.001*E* − 10	1.920*E* − 14	5.020*E* − 08	2.166*E* − 15	4.831*E* − 18	0.000*E* + 00

Aldrin	MS	2.446*E* − 3	5.172*E* − 18	8.825*E* − 09	1.741*E* − 18	8.852*E* − 09	1.995*E* − 18	1.412*E* − 11
	ZM	2.412*E* − 3	4.755*E* − 18	8.492*E* − 09	1.601*E* − 18	8.517*E* − 09	1.835*E* − 18	1.340*E* − 11
	JC	7.304*E* − 4	3.667*E* − 21	3.186*E* − 10	1.235*E* − 21	3.164*E* − 10	1.415*E* − 21	1.542*E* − 13
	TX	2.580*E* − 4	7.123*E* − 24	1.825*E* − 11	2.398*E* − 24	1.797*E* − 11	2.748*E* − 24	3.154*E* − 15

Endrin	MS	8.664*E* − 5	4.575*E* − 25	2.937*E* − 24	3.324*E* − 25	6.513*E* − 24	0.000*E* + 00	6.796*E* − 25
	ZM	1.177*E* − 4	2.876*E* − 24	1.846*E* − 23	2.089*E* − 24	4.094*E* − 23	0.000*E* + 00	4.272*E* − 24
	JC	3.000*E* − 5	7.885*E* − 28	5.062*E* − 27	5.728*E* − 28	1.123*E* − 26	0.000*E* + 00	1.171*E* − 27
	TX	7.348*E* − 5	1.703*E* − 25	1.093*E* − 24	1.237*E* − 25	2.424*E* − 24	0.000*E* + 00	2.529*E* − 25

**Table 7:** The spatial variation of the maximum ecological risk of typical OCPs (PAF).

Pollutant	Site	Max. value (*μ*g/L)	Month	PAF					
				All species	Vertebrates	Invertebrates	Fishes	Crustaceans	Insects and spiders
p,p′-DDT	MS	1.145*E* − 3	8	9.797*E* − 15	6.536*E* − 88	8.683*E* − 11	5.427*E* − 130	9.655*E* − 07	1.537*E* − 71
	ZM	1.067*E* − 3	10	6.248*E* − 15	2.650*E* − 91	6.476*E* − 11	1.628*E* − 135	8.485*E* − 07	2.397*E* − 74
	JC	3.700*E* − 4	10	6.117*E* − 18	2.285*E* − 161	7.220*E* − 13	2.590*E* − 253	1.213*E* − 07	1.100*E* − 132
	TX	4.967*E* − 4	10	4.326*E* − 17	1.040*E* − 137	2.561*E* − 12	7.684*E* − 213	2.086*E* − 07	6.401*E* − 113

*γ*-HCH	MS	1.770*E* − 3	2	1.333*E* − 10	5.887*E* − 08	2.691*E* − 43	4.167*E* − 08	5.447*E* − 79	4.983*E* − 16
	ZM	1.467*E* − 3	2	7.530*E* − 11	4.361*E* − 08	4.212*E* − 47	3.066*E* − 08	4.093*E* − 87	1.889*E* − 16
	JC	2.833*E* − 4	6	4.853*E* − 13	3.145*E* − 09	6.840*E* − 99	2.086*E* − 09	6.763*E* − 206	3.784*E* − 20
	TX	1.053*E* − 3	2	2.738*E* − 11	2.567*E* − 08	1.134*E* − 54	1.783*E* − 08	1.417*E* − 103	3.400*E* − 17

Heptachlor	MS	1.087*E* − 3	2	1.639*E* − 08	2.143*E* − 11	2.110*E* − 06	4.072*E* − 12	4.337*E* − 14	0.000*E* + 00
	ZM	4.867*E* − 4	2	2.598*E* − 09	1.697*E* − 12	5.466*E* − 07	2.672*E* − 13	1.618*E* − 15	0.000*E* + 00
	JC	6.300*E* − 4	1	4.695*E* − 09	3.832*E* − 12	8.435*E* − 07	6.409*E* − 13	4.651*E* − 15	0.000*E* + 00
	TX	4.400*E* − 4	1	2.062*E* − 09	1.235*E* − 12	4.613*E* − 07	1.898*E* − 13	1.070*E* − 15	0.000*E* + 00

Aldrin	MS	1.201*E* − 2	9	7.246*E* − 14	6.996*E* − 07	2.440*E* − 14	7.111*E* − 07	2.796*E* − 14	5.407*E* − 09
	ZM	1.222*E* − 2	9	8.041*E* − 14	7.338*E* − 07	2.707*E* − 14	7.459*E* − 07	3.102*E* − 14	5.769*E* − 09
	JC	2.140*E* − 3	5	2.320*E* − 18	6.112*E* − 09	7.810*E* − 19	6.124*E* − 09	8.948*E* − 19	8.570*E* − 12
	TX	7.313*E* − 3	9	3.694*E* − 15	1.790*E* − 07	1.244*E* − 15	1.812*E* − 07	1.425*E* − 15	8.466*E* − 10

Endrin	MS	3.225*E* − 4	7	1.217*E* − 21	7.812*E* − 21	8.840*E* − 22	1.732*E* − 20	0.000*E* + 00	1.808*E* − 21
	ZM	3.667*E* − 4	5	2.630*E* − 21	1.688*E* − 20	1.911*E* − 21	3.744*E* − 20	0.000*E* + 00	3.907*E* − 21
	JC	3.000*E* − 5	10	7.885*E* − 28	5.062*E* − 27	5.728*E* − 28	1.123*E* − 26	0.000*E* + 00	1.171*E* − 27
	TX	9.000*E* − 5	9	5.748*E* − 25	3.690*E* − 24	4.176*E* − 25	8.183*E* − 24	0.000*E* + 00	8.539*E* − 25

**Table 8:** The spatial and temporary variation of combining ecological risks (msPAF).

Site	Month	All species	Vertebrates	Invertebrates	Fishes	Crustaceans	Insects and spiders
MS	2010.5	1.926*E* − 13	1.011*E* − 08	0.000*E* + 00	9.464*E* − 09	0.000*E* + 00	1.270*E* − 11
	2010.6	1.414*E* − 13	1.263*E* − 08	0.000*E* + 00	1.210*E* − 08	0.000*E* + 00	1.899*E* − 11
	2010.7	1.449*E* − 11	2.659*E* − 08	1.192*E* − 08	2.576*E* − 08	1.954*E* − 08	5.418*E* − 11
	2010.8	5.499*E* − 09	3.986*E* − 09	9.473*E* − 07	3.632*E* − 09	9.655*E* − 07	3.243*E* − 12
	2010.9	3.067*E* − 11	7.012*E* − 07	2.099*E* − 08	7.121*E* − 07	2.798*E* − 14	5.407*E* − 09
	2010.10	5.895*E* − 11	1.259*E* − 08	3.408*E* − 08	1.239*E* − 08	6.597*E* − 07	2.120*E* − 11
	2010.11	5.894*E* − 11	4.801*E* − 10	3.404*E* − 08	3.270*E* − 10	1.316*E* − 07	1.732*E* − 14
	2010.12	1.728*E* − 12	3.372*E* − 09	2.555*E* − 09	3.274*E* − 09	4.271*E* − 08	3.417*E* − 12
	2011.1	1.689*E* − 09	9.302*E* − 09	3.986*E* − 07	8.156*E* − 09	0.000*E* + 00	8.082*E* − 12
	2011.2	1.652*E* − 08	1.056*E* − 07	2.109*E* − 06	8.880*E* − 08	2.892*E* − 08	1.364*E* − 10
	**Mean**	1.584**E** − 10	1.054**E** − 08	7.024**E** − 08	9.976**E** − 09	1.128**E** − 07	1.412**E** − 11

ZM	2010.5	4.681*E* − 13	6.548*E* − 09	0.000*E* + 00	5.509*E* − 09	0.000*E* + 00	3.955*E* − 12
	2010.6	7.053*E* − 12	9.245*E* − 09	7.078*E* − 09	8.706*E* − 09	0.000*E* + 00	1.159*E* − 11
	2010.7	3.411*E* − 13	9.264*E* − 08	0.000*E* + 00	9.266*E* − 08	8.477*E* − 09	3.325*E* − 10
	2010.8	1.130*E* − 13	2.610*E* − 08	0.000*E* + 00	2.574*E* − 08	0.000*E* + 00	5.703*E* − 11
	2010.9	3.008*E* − 10	7.356*E* − 07	1.125*E* − 07	7.473*E* − 07	2.788*E* − 08	5.775*E* − 09
	2010.10	4.374*E* − 14	6.094*E* − 08	6.467*E* − 11	6.118*E* − 08	8.481*E* − 07	1.920*E* − 10
	2010.11	3.754*E* − 11	2.460*E* − 10	2.446*E* − 08	1.563*E* − 10	1.884*E* − 07	0.000*E* + 00
	2010.12	2.436*E* − 10	1.099*E* − 09	9.637*E* − 08	9.890*E* − 10	5.187*E* − 08	5.504*E* − 13
	2011.1	1.444*E* − 09	4.838*E* − 09	3.553*E* − 07	3.832*E* − 09	0.000*E* + 00	1.708*E* − 12
	2011.2	2.673*E* − 09	1.249*E* − 07	5.465*E* − 07	1.127*E* − 07	1.076*E* − 07	2.893*E* − 10
	**Mean**	2.038**E** − 10	1.017**E** − 08	8.451**E** − 08	9.613**E** − 09	7.730**E** − 08	1.340**E** − 11

JC	2010.5	1.744*E* − 13	7.963*E* − 09	0.000*E* + 00	7.338*E* − 09	0.000*E* + 00	8.570*E* − 12
	2010.6	4.855*E* − 13	3.589*E* − 09	0.000*E* + 00	2.527*E* − 09	0.000*E* + 00	2.413*E* − 13
	2010.7	3.836*E* − 13	3.308*E* − 09	0.000*E* + 00	2.362*E* − 09	8.930*E* − 09	3.029*E* − 13
	2010.8	7.927*E* − 14	4.147*E* − 09	0.000*E* + 00	3.716*E* − 09	0.000*E* + 00	3.134*E* − 12
	2010.9	4.730*E* − 14	2.677*E* − 09	0.000*E* + 00	2.340*E* − 09	0.000*E* + 00	1.546*E* − 12
	2010.10	2.770*E* − 10	1.692*E* − 09	1.059*E* − 07	1.536*E* − 09	1.213*E* − 07	1.017*E* − 12
	2010.11	1.116*E* − 10	3.722*E* − 10	5.439*E* − 08	2.388*E* − 10	7.582*E* − 08	0.000*E* + 00
	2010.12	4.545*E* − 11	4.205*E* − 10	2.814*E* − 08	2.681*E* − 10	6.636*E* − 08	0.000*E* + 00
	2011.1	4.695*E* − 09	2.838*E* − 09	8.435*E* − 07	1.923*E* − 09	4.663*E* − 15	5.462*E* − 14
	2011.2	8.353*E* − 10	2.243*E* − 09	2.378*E* − 07	1.538*E* − 09	4.654*E* − 08	7.860*E* − 14
	**Mean**	3.536**E** − 10	1.524**E** − 09	1.266**E** − 07	1.099**E** − 09	4.799**E** − 08	1.542**E** − 13

TX	2010.9	2.189*E* − 11	1.822*E* − 07	1.620*E* − 08	1.833*E* − 07	0.000*E* + 00	8.468*E* − 10
	2010.10	9.437*E* − 14	8.476*E* − 09	2.560*E* − 12	8.024*E* − 09	2.086*E* − 07	1.057*E* − 11
	2010.11	4.774*E* − 15	6.127*E* − 10	1.243*E* − 13	5.042*E* − 10	5.747*E* − 08	1.592*E* − 13
	2010.12	2.461*E* − 12	2.107*E* − 09	3.311*E* − 09	1.980*E* − 09	7.582*E* − 08	1.589*E* − 12
	2011.1	2.068*E* − 09	6.356*E* − 08	4.614*E* − 07	6.032*E* − 08	1.692*E* − 07	1.575*E* − 10
	2011.2	9.511*E* − 10	5.529*E* − 08	2.561*E* − 07	4.766*E* − 08	9.465*E* − 08	7.329*E* − 11
	**Mean**	1.003**E** − 10	2.244**E** − 09	5.021**E** − 08	1.483**E** − 09	1.078**E** − 07	3.109**E** − 15
